# A Machine Learning Approach to Differentiate Congenital and Transient Neonatal Hyperammonemia: A 10-Year Case Series

**DOI:** 10.7759/cureus.98669

**Published:** 2025-12-07

**Authors:** Natalia Frankevich, Alisa Tokareva, Mzia Makieva, Olga Mikhailova, Elena Akhapkina, Vladimir Frankevich

**Affiliations:** 1 Obstetrics and Gynecology, National Medical Research Center for Obstetrics, Gynecology, and Perinatology Named After Academician V.I. Kulakov, Ministry of Health of the Russian Federation, Moscow, RUS; 2 Systems Biology, National Medical Research Center for Obstetrics, Gynecology, and Perinatology Named After Academician V.I. Kulakov, Ministry of Health of the Russian Federation, Moscow, RUS; 3 Neonatology, National Medical Research Center for Obstetrics, Gynecology, and Perinatology Named After Academician V.I. Kulakov, Ministry of Health of the Russian Federation, Moscow, RUS; 4 Translational Medicine, Laboratory of Translational Medicine, Siberian State Medical University, Tomsk, RUS

**Keywords:** ammonia, decision tree, differential diagnosis of hyperammonemia, inborn errors of metabolism, machine learning, neonatal hyperammonemia, predictors of mortality, transient hyperammonemia, urea cycle disorders

## Abstract

Elevated blood ammonia concentration, resulting from various hereditary and acquired conditions, can cause severe damage to the central nervous system, leading to increased rates of disability and infant mortality. In newborns, hyperammonemia is etiologically classified into two main categories: congenital, associated with inborn errors of the urea cycle or organic acidemias, and acquired, which arises secondary to other pathological conditions such as severe perinatal hypoxia, renal or hepatic failure, or intrauterine infections. Despite the differing etiologies, the clinical presentation is often non-specific and may include lethargy, hypotonia, feeding difficulties, respiratory distress, and seizures. This non-specificity frequently leads to initial misdiagnosis. Consequently, a thorough understanding of the pathogenesis, clinical features, and differential diagnosis of congenital versus acquired hyperammonemia is critical for pediatricians, neonatologists, and intensive care specialists. Timely initiation of treatment is paramount, as it directly impacts patient survival and long-term neurological outcomes. Our findings underscore the utility of machine learning in the early differential diagnosis of neonatal hyperammonemia, identifying key predictors that can guide clinical decision-making.

## Introduction

Inborn errors of metabolism (IEMs) caused by enzymatic defects that disrupt urea cycle activity are a primary cause of hyperammonemia in the neonatal period [1-3]. These disorders primarily include congenital urea cycle disorders, classic organic acidemias, and defects in mitochondrial fatty acid oxidation. Conversely, acquired or transient neonatal hyperammonemia (HA) can develop in children without underlying genetic diseases. Risk factors for transient HA include prematurity, hypoxic central nervous system (CNS) injury, infectious complications, liver and kidney diseases, and specific treatments administered to this patient population [4,5].

Differential diagnosis is particularly challenging between conditions presenting with hyperammonemia, such as neonatal sepsis, and the initial manifestation of an IEM in the newborn period. The initial clinical signs of HA are non-specific, complicating recognition and potentially leading to delayed treatment initiation and irreversible CNS damage.

Elevated blood ammonia concentration, resulting from various hereditary or acquired conditions, causes significant CNS injury through its toxic effects on astrocytes. Consequently, patients with hyperammonemia exhibit potentially life-threatening neuropsychiatric symptoms, the severity of which depends on the magnitude and duration of hyperammonemia, as well as the brain's developmental stage. The neurotoxicity of ammonia remains a critical challenge in neonatology; ammonia is a potent neurotoxin, and levels exceeding the normal range can lead to rapid brain injury. Modern neuroimaging techniques can detect cerebral metabolic disturbances even before structural changes become apparent [6]. Neonates with severe hyperammonemia often exhibit findings such as cortical atrophy, ventricular dilatation, and demyelination, which lead to severe cognitive impairments and cerebral palsy [7,8].

Long-term follow-up studies confirm that even brief episodes of hyperammonemia can result in neurocognitive developmental deficits, including impairments in executive function and memory [9]. International consensus guidelines recommend considering hyperammonemia in the differential diagnosis of all neonatal encephalopathies, particularly in cases of refractory seizures, progressive CNS depression, a discrepancy between clinical severity and laboratory parameters, or lack of response to standard therapy [10].

Early detection and management of hyperammonemia are crucial for patient survival and improving outcomes. HA is a potentially lethal condition, necessitating the immediate treatment of acute episodes to prevent irreversible neurological damage, even before a definitive diagnosis is established.

The early diagnosis of congenital metabolic disorders leading to hyperammonemia involves measuring metabolites in biological fluids, and sometimes requires rapid enzymatic assays and molecular genetic testing. While the ammonia level is critical for diagnosis, its measurement must be prioritized and precede other specialized laboratory investigations. Expanding the neonatal screening panel for IEMs and implementing universal blood ammonia monitoring in high-risk newborn populations could become a valuable future strategy for the early identification of infants at high risk for HA and the timely initiation of therapy.

The objective of this study was to characterize cases of neonatal hyperammonemia, utilizing machine learning methods, in patients with inherited metabolic diseases presenting in the neonatal period and those with transient forms of this complication, all requiring admission to the pediatric intensive care unit at the V.I. Kulakov National Medical Research Center for Obstetrics, Gynecology and Perinatology, Moscow, Russia, between 2017 and 2025.

## Materials and methods

An observational study with retrospective data collection was conducted, including neonates with HA admitted to the Pediatric Intensive Care Unit of V.I. Kulakov National Medical Research Center for Obstetrics, Gynecology and Perinatology, Moscow, Russia, between June 2017 and June 2025. Case identification was performed using the hospital's unified electronic database of primary medical records. The study was approved by the Ethics Committee of the V.I. Kulakov National Medical Research Center for Obstetrics, Gynecology and Perinatology (protocol number: 4, dated April 24, 2025). Written informed consent was obtained from all participants, and the study protocol (No. 4, 24 April 2025) was approved by the IRB of the Kulakov's Center.

Study population

The study initially considered 48 neonates. Following a review of the primary medical records, 15 cases were excluded from the analysis. The exclusions were as follows: five cases due to a lack of confirmatory data for an IEM diagnosis, seven cases due to incomplete laboratory datasets required for analysis (resulting from the patient's transfer to another medical institution), and three cases due to the unavailability of autopsy reports (as the children were transferred to other facilities prior to death). The number of neonatal in the final cohort was 33. Two pathophysiological groups were defined: cases of HA caused by the onset of an IEM (n=12) and cases with transient HA (n=21).

Investigations

All patients included in the study underwent a comprehensive diagnostic workup in the intensive care unit, including complete blood count, urinalysis, comprehensive biochemical blood analysis, determination of systemic inflammatory response markers, echocardiography, neurosonography, abdominal ultrasonography, and chest radiography.

Plasma ammonia concentration was measured spectrophotometrically using the Ammonia Ultra kit (Sentinel Diagnostics - Sentinel CH. S.p.A., Milan, Italy) according to the manufacturer's protocol. Pathological blood ammonia levels were defined as >100 μmol/L in term neonates and >150 μmol/L in preterm neonates. Indications for blood ammonia testing included clinical deterioration manifested by increasing signs of CNS depression, episodes of apnea, development of seizures of unknown origin, or clinical signs of sepsis in the absence of laboratory markers of systemic inflammatory response. In one case, blood ammonia monitoring was initiated from birth due to a high risk of developing an IEM (the first child in the family had died from an IEM).

In our cohort, all neonates with confirmed hyperammonemia underwent specific metabolic investigation. First-line tests for suspected IEM included blood tests for ammonia, lactate, pyruvate, uric acid, urea, cholesterol, creatine phosphokinase, triglycerides, liver function tests, and coagulation profile; urinalysis for ketone bodies and pH. It is noteworthy that IEMs are often characterized by specific urine odor and color. Second-line investigations included blood tests for amino acid and acylcarnitine profiles, urine tests for organic acid profile, and orotic acid concentration.

For all children with identified hyperammonemia, enteral feeding and intravenous protein administration were discontinued. The period of protein restriction was accompanied by increased parenteral administration of carbohydrates and lipids to meet energy requirements and prevent catabolic processes. The duration of protein restriction ranged from one to three days, with gradual reintroduction of protein as blood ammonia levels normalized.

Data analysis

Using machine learning methods, an analysis was performed to identify factors significantly associated with the type of HA (congenital or transient) and patient outcome (survived or deceased). Clinical parameters were compared based on the etiology of HA and based on outcomes (14 cases resulted in a fatal outcome). Continuous clinical parameters were compared using the Mann-Whitney U test, while categorical parameters were compared using Pearson's chi-square test. Continuous clinical parameters are presented as median (first quartile; third quartile), and categorical parameters as absolute values (percentage within group). Differences with p < 0.05 were considered statistically significant. Symptoms and anamnestic data showing statistically significant differences were incorporated into decision trees for differential diagnosis of HA etiology and for identifying patients at high risk of mortality.

Model quality was assessed using 10-fold cross-validation with calculation of accuracy, sensitivity, and specificity. All computations were performed using R language version 4.3.3 (R Foundation for Statistical Computing, Vienna, Austria, https://www.R-project.org/)[11], with the packages rpart 4.1.24 [12] for decision trees, pROC 1.18.5 [13] for ROC analysis, effsize 0.8.1 [14] for effect size calculation, pwr 1.3-0 [15] for power analysis, and ggplot2 3.5.2 [16] and rpart.plot 3.1.12 [17] for result visualization.

## Results

The final study cohort included 15 term neonates (n=6, 50% and n=9, 43%, in the congenital and transient HA groups, respectively) and 18 preterm neonates (n=6, 50% and n=12, 57%, in the congenital and transient HA groups, respectively). The distribution of preterm neonates by gestational age category in the two groups (congenital HA and transient HA) was as follows: extremely preterm: 1 (8%) and 6 (29%); very preterm: 1 (8%) and 5 (24%); moderate preterm: 1 (8%) and 1 (5%); and late preterm: 3 (25%) and 0 (0%), respectively. The median gestational age at delivery was 36.5 weeks (IQR: 33.5, 38) and 30.6 weeks (IQR: 27, 38) in the congenital and transient HA groups, respectively. The corresponding median birth weights were 2268.5 g (IQR: 1715.75, 3369.5) and 1100 g (IQR: 780, 2990). The median Apgar score at one minute was 6 (range, 1-8) and at five minutes was 7 (range, 3-9). Delivery was via cesarean section for 25 (75%) neonates and vaginal for eight (15%) neonates. The cohort included eight (24%) children from multiple pregnancies and 14 boys (42%) and 19 girls (58%).

**Figure 1:**
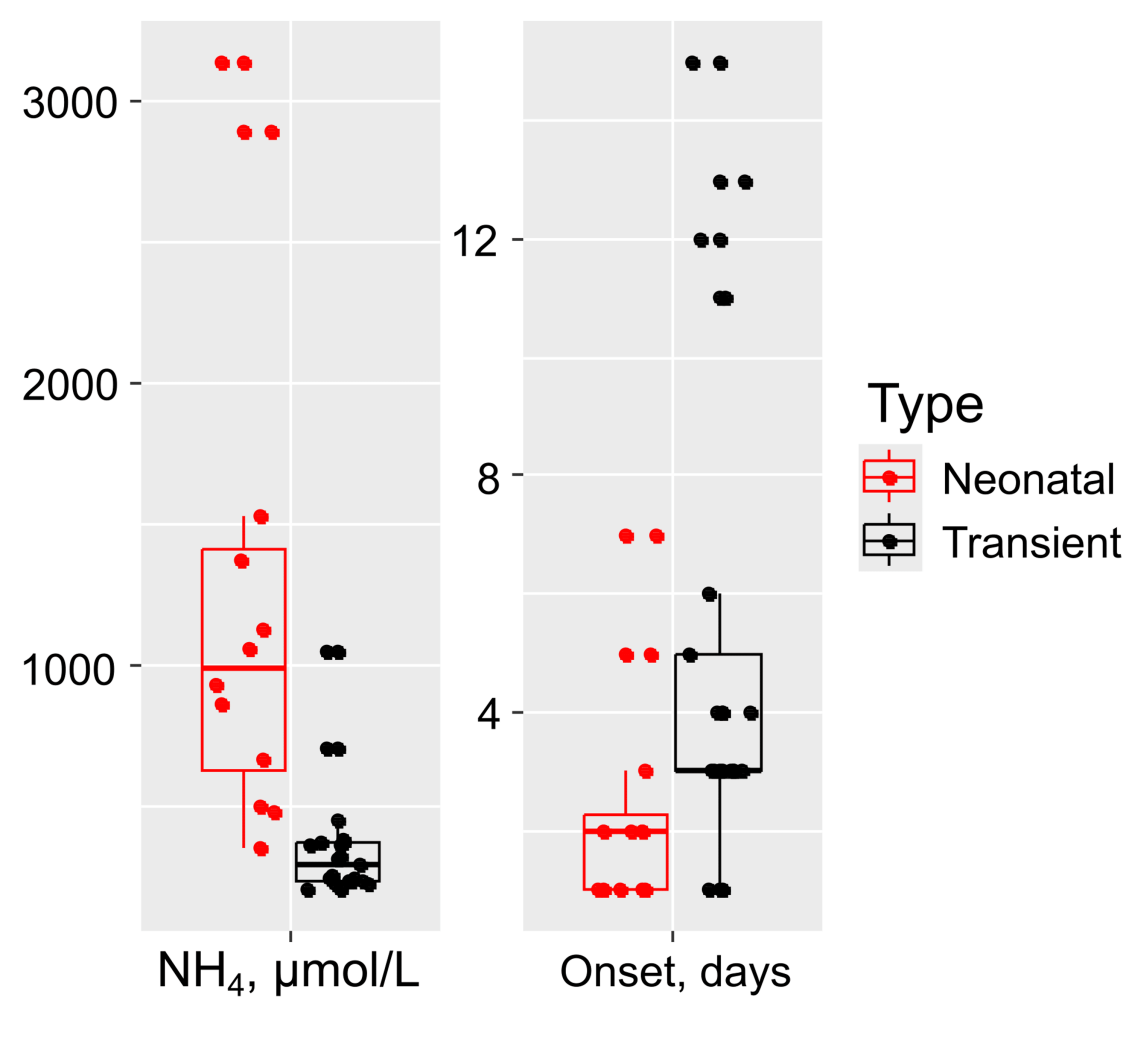
Statistically significant differences in the onset day and peak ammonia level between congenital and transient hyperammonemia groups

A history of aciduria in family members was reported exclusively in the group with HA caused by IEM onset (n=4, 33%, p=0.02). The onset of HA occurred significantly earlier in the congenital HA group (2 (1; 2.25) days) compared to the transient HA group (3 (3; 5) days), (p = 0.01). Furthermore, the peak blood ammonia concentration was significantly higher in the congenital HA group (median 995.2 µmol/L (IQR: 628.15, 1409.25)) compared to the transient HA group (median 295 µmol/L (IQR: 236.4, 376)), (p < 0.001) (Figure 1).

A fatal outcome was documented in eight (67%) children in the congenital HA group and in six (29%) in the transient HA group. A detailed characterization of the groups is presented in Table 1.

**Table 1:** Clinical parameters of infants with hyperammonemia according to the type of hyperammonemia, statistical probability of sample matching, and test power. IEM: inborn errors of metabolism; IUGR: intrauterine growth restriction; IVH: intraventricular hemorrhage; CBC: complete blood count

Clinical parameters	Congenital HA (IEM) (n=12), n (%)	Transient HA (n=21), n (%)	p-Value	Test power
Gestational age at delivery (weeks), n (%)				
Extremely preterm (<28 weeks)	1 (8%)	6 (29%)	0.09	0.60
Very preterm (28 - <32 weeks)	1 (8%)	5 (24%)		
Moderate preterm (32 - <34 weeks)	1 (8%)	1 (5%)		
Late preterm (34 - <37 weeks)	3 (25%)	0 (0%)		
Term (≥37 weeks)	6 (50%)	9 (43%)		
Gestational age at delivery (weeks), median (IQR)	36.5 (33.5, 38)	30.6 (27, 38)	0.39	0.24
Birth weight (g), median (IQR)	2268.5 (1715.75, 3369.5)	1100 (780, 2990)	0.08	0.35
IUGR, n (%)	4 (33%)	9 (43%)	0.87	0.05
Severe birth asphyxia, n (%)	6 (50%)	17 (81%)	0.14	0.31
Pneumonia, n (%)				
Congenital	7 (58%)	15 (71%)	0.07	0.53
Acquired	1 (8%)	5 (24%)		
Absent	4 (33%)	1 (5%)		
Sepsis, n (%)	2 (17%)	5 (24%)	0.97	0.05
Valproic acid treatment, n (%)	8 (67%)	12 (57%)	0.87	0.05
IVH, n (%)	4 (33%)	11 (52%)	0.49	0.11
Hyperbilirubinemia, n (%)	8 (67%)	15 (71%)	1.00	0.05
Anemia, n (%)	9 (75%)	17 (81%)	1.00	0.05
Congenital malformations, n (%)	2 (17%)	3 (14%)	1.00	0.05
Male sex, n (%)	7 (58%)	7 (33%)	0.30	0.18
Pregnancy number, median (IQR)	2 (1; 3.5)	2 (2; 4)	0.45	0.08
Pregnancy complications, n (%)				
None	4 (33%)	7 (33%)	0.19	0.53
Feto-fetal transfusion syndrome	0 (0%)	7 (33%)		
Premature rupture of membranes	3 (25%)	3 (14%)		
Preeclampsia	2 (17%)	1 (5%)		
Fetal growth restriction	3 (25%)	2 (10%)		
Genital herpes-associated infections	0 (0%)	1 (5%)		
Family history of aciduria, n (%)	4 (33%)	0 (0%)	0.02	0.62
Invasive respiratory therapy, n (%)	10 (83%)	16 (76%)	0.97	0.05
Non-invasive respiratory therapy, n (%)	8 (67%)	15 (71%)	1.00	0.05
Inotropic/vasopressor support, n (%)	11 (92%)	17 (81%)	0.75	0.06
Renal failure, n (%)	7 (58%)	8 (38%)	0.45	0.12
Onset of hyperammonemia (days), median (IQR)	2 (1; 2.25)	3 (3; 5)	0.01	0.57
Hypoglycemia, n (%)	2 (17%)	5 (24%)	0.97	0.05
Seizures, n (%)	8 (67%)	12 (57%)	0.87	0.05
CBC, median (IQR)				
Leukocytes (×10⁹/L)	12.45 (6.4, 16.25)	15.79 (13.01, 22.23)	0.13	0.17
Neutrophils	6711 (2994.25, 10309.25)	7622 (3036, 10532)	0.90	0.05
Platelets (×10⁹/L)	261 (208.75, 281.5)	182 (139, 330)	0.20	0.18
Erythrocytes (×10¹²/L)	4.8 (4.22, 4.94)	4.15 (3.74, 4.97)	0.26	0.19
Hemoglobin (g/L)	171.5 (157.75, 184)	157 (141, 180)	0.20	0.18
Hematocrit	0.49 (0.44, 0.53)	0.47 (0.43, 0.51)	0.47	0.09
NH_4_^+^ at disease onset (µmol/L)	995.2 (628.15, 1409.25)	295 (236.4, 376)	<0.001	0.99
Urea (mmol/L)	3.8 (2.58; 6.7)	6.2 (4.2; 9.8)	0.22	0.13
Creatinine (µmol/L)	70.05 (62.9, 99.28)	88.4 (70.2, 106.1)	0.52	0.14
Calcium (mmol/L)	2.32 (2.07; 2.54)	2.24 (2.07; 2.36)	0.65	0.11
Fatal outcome, n (%)	8 (67%)	6 (29%)	0.08	0.42

For a detailed analysis of factors associated with patient outcomes (survived or deceased), the cohort was subsequently stratified into two subgroups: Subgroup A included 19 children with a favorable outcome (survived), and Subgroup B included 14 children with a lethal outcome. The incidence of renal failure was statistically significantly lower in Subgroup A (n=3, 16%) compared to the fatal outcome group (n=12, 86%), p < 0.001. Children with a favorable outcome received non-invasive respiratory therapy significantly more often (n=17 (89%) vs. n=6 (43%), respectively, p = 0.01).

**Figure 2:**
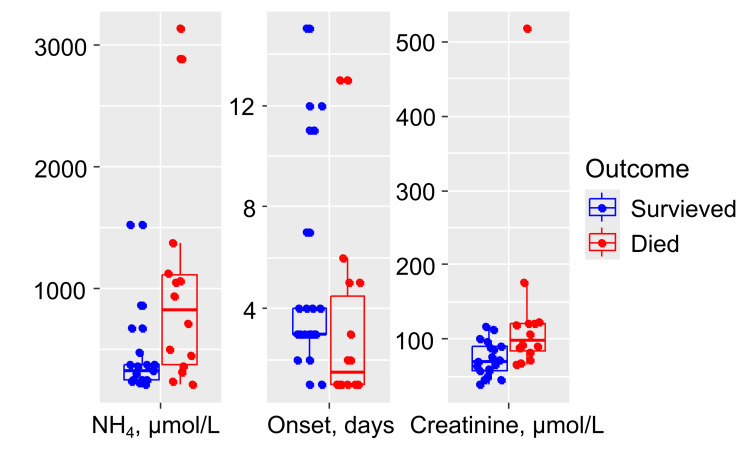
Markers demonstrating statistically significant differences based on the outcomes of hyperammonemia

In children with a fatal outcome, the peak recorded blood ammonia level (median 819.7 µmol/L (IQR: 379.25; 1107.5)) and the creatinine level on the first day of life (median 99.25 µmol/L (IQR: 83.6, 121.82)) were significantly higher (p = 0.02 and p = 0.002, respectively) than in the group of survivors (median 326 µmol/L (IQR: 243.25, 378.5) and 70.2 µmol/L (IQR: 57.45; 89.55)) (Figure 2). This reflects the earlier onset and greater severity of hyperammonemia in the subgroup with a lethal outcome. A detailed characterization of subgroups A and B is presented in Table 2.

**Table 2:** Clinical parameters of newborns with hyperammonemia stratified by disease outcome, statistical probability of sample matching, and test power IEM: inborn errors of metabolism; IUGR: intrauterine growth restriction; IVH: intraventricular hemorrhage; CBC: complete blood count

Clinical Parameters	Survived (n=19)	Died (n=14)	p-Value	Test Power
Type of HA, n (%)				
Transient HA	15 (79%)	6 (43%)	0.08	0.42
Neonatal HA (IEM)	4 (21%)	8 (57%)		
Gestational age at delivery (weeks), n (%)				
Extremely preterm (<28 weeks)	3 (16%)	4 (29%)	0.39	0.33
Very preterm (28 - <32 weeks)	4 (21%)	2 (14%)		
Moderate preterm (32 - <34 weeks)	0 (0%)	2 (14%)		
Late preterm (34 - <37 weeks)	2 (11%)	1 (7%)		
Term (≥37 weeks)	10 (53%)	5 (36%)		
Gestational age at delivery (weeks), median (IQR)	37 (29.2, 39)	32 (27.25, 37)	0.13	0.30
Birth weight (g), median (IQR)	2210 (1098, 3056.5)	1231.5 (780, 2926.25)	0.28	0.17
IUGR, n (%)	7 (37%)	6 (43%)	1.00	0.05
Severe birth asphyxia, n (%)	13 (68%)	10 (71%)	1.00	0.05
Pneumonia, n (%)				
Congenital	14 (74%)	8 (57%)	0.18	0.36
Acquired	4 (21%)	2 (14%)		
Absent	1 (5%)	4 (29%)		
Sepsis, n (%)	2 (11%)	5 (36%)	0.19	0.26
Valproic acid treatment, n (%)	10 (53%)	10 (71%)	0.46	0.11
IVH, n (%)	6 (32%)	9 (64%)	0.13	0.33
Hyperbilirubinemia, n (%)	13 (68%)	10 (71%)	1.00	0.05
Anemia, n (%)	14 (74%)	12 (86%)	0.69	0.07
Congenital malformations, n (%)	4 (21%)	1 (7%)	0.54	0.09
Male sex, n (%)	10 (53%)	4 (29%)	0.30	0.18
Pregnancy number, median (IQR)	2 (1, 3)	2.5 (1.25, 4.75)	0.56	0.05
Pregnancy complications, n (%)				
None	7 (37%)	4 (29%)	0.78	0.19
Feto-fetal transfusion syndrome	3 (16%)	4 (29%)		
Premature rupture of membranes	4 (21%)	2 (14%)		
Preeclampsia	1 (5%)	2 (14%)		
Fetal growth restriction	3 (16%)	2 (14%)		
Other	1 (5%)	0 (0%)		
Family history of aciduria, n (%)	1 (5%)	3 (21%)	0.39	0.14
Invasive respiratory therapy, n (%)	14 (74%)	12 (86%)	0.69	0.07
Non-invasive respiratory therapy, n (%)	17 (89%)	6 (43%)	0.01	0.70
Inotropic/vasopressor support, n (%)	15 (79%)	13 (93%)	0.54	0.09
Renal failure, n (%)	3 (16%)	12 (86%)	<0.001	0.95
Onset of hyperammonemia (days), median (IQR)	3 (3, 4)	1.5 (1, 4.5)	0.04	0.26
Hypoglycemia, n (%)	2 (11%)	5 (36%)	0.19	0.26
Seizures, n (%)	10 (53%)	10 (71%)	0.46	0.11
CBC, median (IQR)				
Leukocytes (×10⁹/L)	14.44 (13.02, 20.23)	13.4 (7.64, 20.23)	0.53	0.05
Neutrophils (cells)	7622 (5266, 9813.5)	6458 (1471.25, 11424.5)	0.42	0.07
Platelets (×10⁹/L)	215 (176, 338)	216.5 (106.75, 276.25)	0.30	0.21
Erythrocytes (×10¹²/L)	4.74 (4.04, 4.97)	4.43 (3.8, 4.9)	0.66	0.07
Hemoglobin (g/L)	160 (153.5, 188.5)	168 (140, 180.75)	0.74	0.05
Hematocrit	0.47 (0.44, 0.53)	0.48 (0.4, 0.52)	0.73	0.07
NH_4_^+^ at disease onset (µmol/L)	326 (243.25, 378.5)	819.7 (379.25, 1107.5)	0.02	0.77
Urea (mmol/L)	4.4 (2.6, 6.95)	7.25 (4.38, 12.6)	0.10	0.36
Creatinine (µmol/L)	70.2 (57.45, 89.55)	99.25 (83.6, 121.82)	0.002	0.58
Calcium (mmol/L)	2.27 (2.15, 2.47)	2.12 (1.98, 2.46)	0.17	0.15

**Figure 3:**
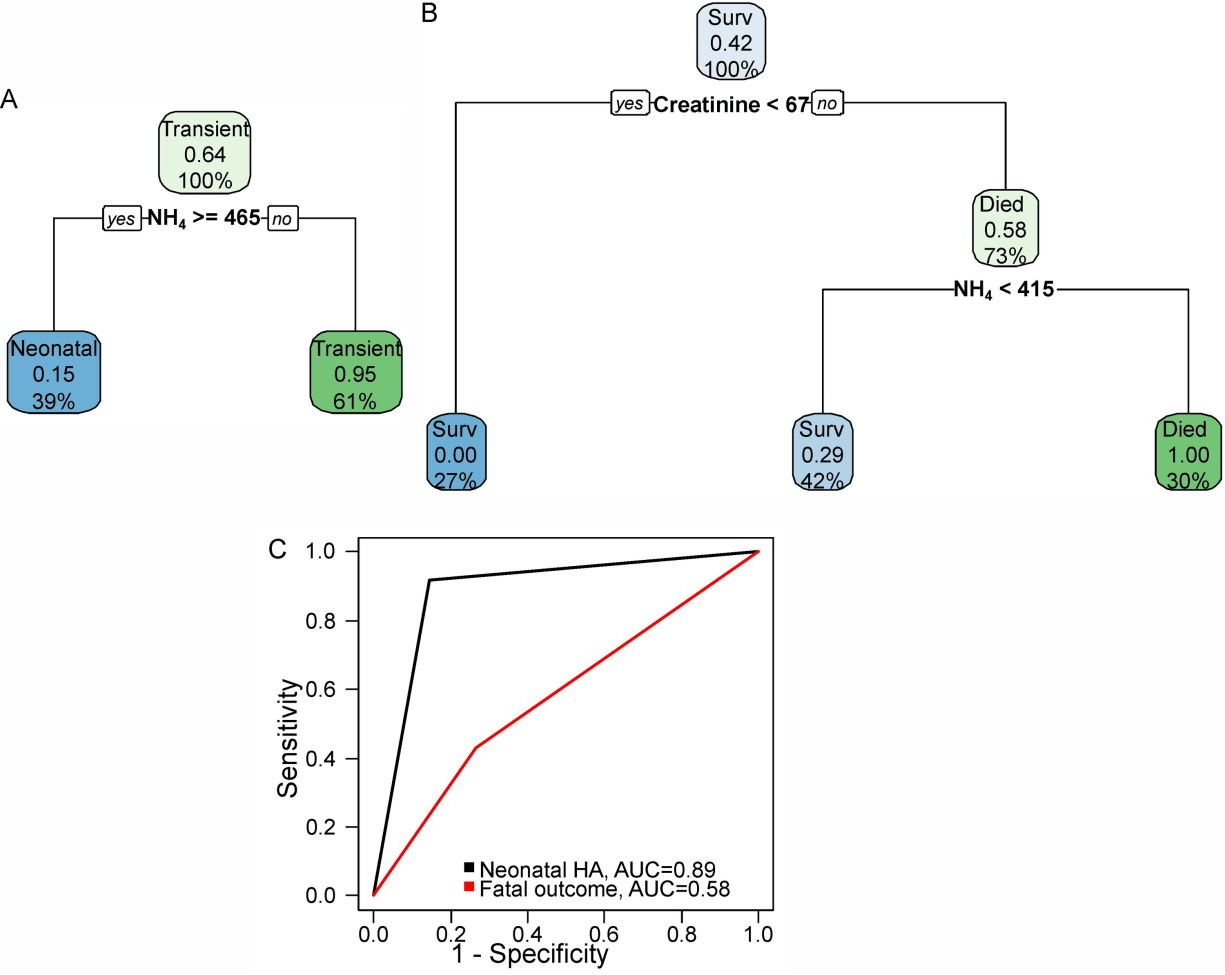
(A) Decision tree for identifying hyperammonemia etiology, (B) Decision tree for identifying the high-risk mortality group, (C) Receiver operating characteristic (ROC) curves obtained during model validation Surv: survived; HA: hyperammonemia; AUC: area under the curve

Following the analysis of the obtained data using machine learning methods, a decision tree was constructed to identify congenital HA (Figure 3a), characterized by a sensitivity of 92%, specificity of 86%, and accuracy of 88% (Figure 3c). Additionally, a decision tree for determining a high risk of fatal outcome in HA (Figure 3b) was built, demonstrating a sensitivity of 43%, specificity of 74%, and accuracy of 61% (Figure 3c). The risk ratio between the high-risk and low-risk groups for fatal outcome was 1.5 (95%CI 0.9 - 2.6).

## Discussion

The primary finding of our study is that a decision tree model based on the day of onset and peak ammonia level can effectively differentiate between congenital and transient HA with high accuracy (88%). During the neonatal period, infants experience a physiological elevation in blood ammonia concentration that can persist for up to one month. This transient state is driven by the activation of catabolic processes necessary for the redistribution of nutrients to organs, facilitating adaptation to extrauterine life [1,18]. However, in the presence of inherited metabolic disorders or complicating factors, this physiological state can escalate into life-threatening pathological HA. The early detection and management of this condition are therefore critical for patient survival and outcome improvement.

Congenital HA is associated with a broad spectrum of genetic disorders, including urea cycle defects, organic acidemias, fatty acid oxidation disorders, and other metabolic disturbances [9]. Recent investigations further highlight the role of epigenetic factors in modulating the phenotypic severity of these conditions [19]. The primary risk factors for transient HA include extreme prematurity (due to functional immaturity of hepatic enzyme systems), perinatal asphyxia, ongoing infectious processes, and specific pharmacological exposures, particularly valproic acid therapy [20,21]. Contemporary research demonstrates that hypoxic-ischemic injury triggers a cascade of molecular events, including oxidative stress and mitochondrial dysfunction, which exacerbates the impairment of ammonia detoxification [22].

Given the non-specific nature of the initial symptoms, HA should be considered in the differential diagnosis of newborns presenting with neurological impairments of varying severity, respiratory distress, muscular hypotonia, as well as in cases where the clinical picture suggests sepsis but lacks supporting markers of systemic inflammatory response, or when there is a lack of response to conventional therapy [23].

The diagnostic capabilities of neonatal screening in Russia had achieved significant progress by 2025, with the program expanded to include 36 hereditary diseases (in accordance with the Russian Ministry of Health Order No. 274н dated April 21, 2022), 12 of which are nosologies associated with a risk of developing HA.

Significant progress has been made in the field of predictive diagnostics. Studies have reported the development of multifactorial mathematical models for assessing the risk of HA development and fatal outcomes [24-26]. These models incorporate various parameters, including patient history, anthropometric measurements, laboratory results, and markers of hypoxic-ischemic injury.

Analysis of our study data using machine learning methods enabled the creation of a decision tree for differentiating between transient HA and HA caused by IEM onset. This model demonstrated a sensitivity of 92%, specificity of 86%, and accuracy of 88%. The two most significant discriminatory markers were the timing of HA onset (day of life) and the blood ammonia concentration (μmol/L). The initial blood ammonia level not only helps differentiate the type of HA but also serves as a key predictive marker for mortality. Supporting this, a research team from Nizhny Novgorod in 2021 developed a diagnostic model to predict the likelihood of IEM in neonates presenting with HA syndrome and to determine appropriate management strategies [25]. Their discriminant analysis established a statistically significant association (p=0.040) between the risk of IEM and the initial ammonia level, blood glucose, base deficit, lactate, hemoglobin, erythrocyte count, mean corpuscular volume, and pH. Their model achieved a sensitivity of 87.5%, specificity of 83.3%, and diagnostic accuracy of 85.0%.

In our study, the primary significant predictors of mortality were the initial blood ammonia level and the blood creatinine level on the first day of life. Given the high neurotoxicity of ammonia, its level serves as an independent prognostic factor for in-hospital mortality, a finding also observed in adult patients [25]. A model developed in 2021 demonstrated 94.2% sensitivity and 88.7% specificity in predicting the risk of pathological HA within the first 72 hours of life [26].

The decision tree we developed for identifying a high risk of mortality in HA showed a sensitivity of 43%, specificity of 74%, and accuracy of 61%. The risk ratio for mortality among children with median blood ammonia levels of 819.7 μmol/L (IQR: 379.25, 1107.5) and median first-day creatinine levels of 99.25 μmol/L (IQR: 83.6, 121.82), compared to children with lower values (median 326 μmol/L (IQR: 243.25, 378.5) μmol/L and 70.2 μmol/L (IQR: 57.45, 89.55), p = 0.02 and p = 0.002, respectively), was 1.5 (95%CI, 0.9 - 2.6).

The expansion of neonatal screening using tandem mass spectrometry has significantly improved the early detection of metabolic disorders. A promising future direction is the integration of whole-genome sequencing into the diagnostic protocols for high-risk newborns [27]. In Russia, a pilot program implementing NGS sequencing for high-risk neonates was launched in 2024 across 15 federal centers [28].

Genetic counseling for families is of paramount importance, as establishing a diagnosis enables the possibility of prenatal diagnosis in subsequent pregnancies. The small sample size is a limitation of the study; however, despite this, the available evidence underscores the critical importance of measuring blood ammonia levels in at-risk children. This test can serve as a specific laboratory tool for the differential diagnosis of inherited metabolic diseases and for prognosticating the risk of mortality.

## Conclusions

Rapid and effective management of HA is crucial for preventing irreversible neurological damage. Current priorities in perinatal medicine necessitate the enhancement of neonatal screening programs and the facilitation of early HA diagnosis within pediatric intensive care settings. Future efforts should focus on integrating the developed predictive models into routine clinical practice, achieving universal coverage of neonatal screening, and advancing telemedicine consultation systems to provide expert support in remote regions.
